# Angiotensin-(1-12): Does It Exist? A Critical Evaluation in Humans, Rats, and Mice

**DOI:** 10.1161/HYPERTENSIONAHA.124.22856

**Published:** 2024-05-08

**Authors:** André F. Rodrigues, Oliver Domenig, Marko Poglitsch, Michael Bader, A.H. Jan Danser

**Affiliations:** 1 Max Delbrück Center for Molecular Medicine in the Helmholtz Association, Berlin, Germany (A.F.R., M.B.).; 2 German Center for Cardiovascular Research, Berlin, Germany (A.F.R., M.B.).; 3 Attoquant Diagnostics, Vienna, Austria (O.D., M.P.).; 4 Charité Universitätsmedizin Berlin, Germany (M.B.).; 5 Institute for Biology, University of Lübeck, Germany (M.B.).; 6 Division of Pharmacology and Vascular Medicine, Department of Internal Medicine, Erasmus MC, University Medical Center Rotterdam, The Netherlands (A.H.J.D.).

**Keywords:** angiotensin II, angiotensinogen, chromatography, liquid, mice, knockout, tandem mass spectrometry

## Abstract

**BACKGROUND:**

Angiotensin-(1-12), measured by a self-developed, polyclonal antibody-based radioimmunoassay, has been suggested to act as an alternative precursor of angiotensin II. A more reliable detection method would be liquid chromatography-tandem mass spectrometry.

**METHODS:**

We set up the quantification of human and murine angiotensin-(1-12) by liquid chromatography-tandem mass spectrometry and then used this method to measure angiotensin-(1-12) in human, rat, and mouse blood samples, as well as in mouse brain, mouse kidney, and rat heart. We also verified ex vivo angiotensin-(1-12) generation and metabolism in human blood samples incubated at 37 °C.

**RESULTS:**

Stabilization of blood in guanidine hydrochloride was chosen for sample collection since this allowed full recovery of spiked angiotensin-(1-12). Angiotensin-(1-12) was undetectable in human blood samples when incubating nonstabilized plasma at 37 °C, while angiotensin-(1-12) added to nonstabilized human plasma disappeared within 10 minutes. Stabilized human blood samples contained angiotensin II, while angiotensin-(1-12) was undetectable. Blood, hearts, and kidneys, but not brains, of wild-type mice and rats contained detectable levels of angiotensin II, while angiotensin-(1-12) was undetectable. In renin knockout mice, all angiotensins, including angiotensin-(1-12), were undetectable at all sites, despite a 50% rise in angiotensinogen. Angiotensin-(1-12) metabolism in human blood plasma was not affected by renin inhibition. Yet, blockade of angiotensin-converting enzyme and aminopeptidase A, but not of chymase, neutral endopeptidase, or prolyl oligopeptidase, prolonged the half-life of angiotensin-(1-12), and angiotensin-converting enzyme inhibition prevented the formation of angiotensin II.

**CONCLUSIONS:**

We were unable to detect intact angiotensin-(1-12) in humans, rats, and mice, either in blood or tissue, suggesting that this metabolite is an unlikely source of endogenous angiotensins.

**NOVELTY AND RELEVANCE**

**What Is New?**

We set up a highly sensitive quantification method for human and murine angiotensin-(1-12), using liquid chromatography-tandem mass spectrometry.

With this method, we were able to fully recover spiked angiotensin-(1-12) added to stabilized human blood samples (ie, blood samples collected in guanidine hydrochloride). However, without spiking, we could not detect angiotensin-(1-12) in such samples, although angiotensin I, angiotensin, renin, and angiotensinogen were readily detectable.

The blood, hearts, and kidneys of wild-type mice and rats also did not contain angiotensin-(1-12), although angiotensins I and II were detectable. In renin knockout mice, all angiotensins, including angiotensin-(1-12), were undetectable, despite a 50% rise in angiotensinogen.

Angiotensin-(1-12) added to human plasma was rapidly metabolized by ACE and aminopeptidase A, while renin, chymase, neutral endopeptidase, and prolyl oligopeptidase did not contribute to its metabolism.

**What Is Relevant?**

Angiotensin-(1-12) is an unlikely source of endogenous angiotensins, neither in the blood nor in tissues.

**Clinical/Pathophysiological Implications?**

Previous studies detecting angiotensin-(1-12) by radioimmunoassay at levels that were 1000-fold higher than our current detection limit most likely measured angiotensinogen, given the cross-reactivity of the polyclonal antibody with this precursor.

Angiotensin-(1-12) immunization is an unlikely tool to treat hypertension, although it may partly suppress angiotensinogen.

Angiotensin-(1-12) (Ang-[1-12]), first described in 2006,^1^ has been suggested to act as an alternative precursor of angiotensin II (Ang II), circumventing the classical renin-dependent pathway.^2^ Both angiotensin-converting enzyme (ACE; extracellular, eg, in blood) and chymase (intracellular, eg, at tissue sites) generate Ang II from Ang-(1-12), implying that Ang II generation would continue in anephric animals.^3^ Yet, others reported undetectable Ang II levels after a bilateral nephrectomy,^4–6^ while trials with chymase inhibitors were negative.^7^ Moreover, the effects of renin inhibitors are comparable to those of other renin-angiotensin system blockers.^8–10^

A self-developed radioimmunoassay yielded blood Ang-(1-12) levels in the order of 2 ng/mL, which corresponds with <0.01% of the levels of angiotensinogen.^11^ Remarkably, the polyclonal antibody used in this assay displayed 3% cross-reactivity with angiotensinogen, implying that the detected Ang-(1-12) levels might still represent angiotensinogen itself. A more reliable detection method to quantify Ang-(1-12), not relying on antibody specificity, would be liquid chromatography-tandem mass spectrometry (LC-MS/MS). This approach makes use of stable isotope-labeled internal standards (IS) of each individual angiotensin metabolite (including Ang-[1-12] itself) for signal normalization, added to each sample before preparation, thus ensuring the highest accuracy and specificity. Angiotensin measurements based on LC-MS/MS yield much lower levels than commercial radioimmunoassays.^12,13^ Although LC-MS/MS has already been used to quantify Ang-(1-12),^14–17^ the currently available data were either obtained without using individual standards (rendering levels that do not necessarily represent Ang-(1-12)^15^) or were accompanied by Ang II levels that were 2 to 3 orders of magnitude higher than normally reported.^14,18–20^ Indeed, the 2 groups^14–17^ that applied LC-MS/MS earlier used Nor-Leu^3^-Ang-(1-7) and [Asn^1^, Val^5^]-Ang II as IS for all angiotensin metabolites in their methods. Retention times for these compounds are significantly different compared with Ang-(1-12), which leaves open the risk of matrix effects on Ang-(1-12) signals that are not picked up by the IS used. Importantly, the availability of stable isotope-labeled peptides has paved the way for the implementation of optimal IS in peptide quantification. The low relative mass difference (<1%) does not affect the HPLC retention time, while the incorporation of a C^13^/N^15^-labeled amino acid does not affect the chemical properties of the peptide, as the electron cloud, being the determinant of a molecule’s chemical properties, remains unaffected by C^13^/N^15^ labeling. This results in identical sample processing recovery and identical chemical reactivity, both being essential for state-of-the-art normalization of LC-MS/MS detection and bio-analytic process quality control. Only the normalization of endogenous Ang-(1-12) signals using stable isotope-labeled Ang-(1-12) as the corresponding IS assures control for all factors that might interfere with the detection of the endogenous peptide. Spiking stable isotope-labeled Ang-(1-12) into samples before analyte extraction allows for recovery control throughout the entire analytical process, down to individual fragmentation reactions in MS/MS detection. Importantly, the absence of a stable isotope-labeled IS signal clearly indicates a problem with the bio-analytical process.

In the present study, we first set up the quantification of human and murine (rat and mouse) Ang-(1-12) by LC-MS/MS, specifically applying stable isotope-labeled IS of each individual angiotensin metabolite, including Ang-(1-12) itself. If the stable isotope-labeled Ang-(1-12) can be detected in a sample while the endogenous Ang-(1-12) signal in the identical sample is absent, this serves as a factual proof of the absence of endogenous Ang-(1-12) in this sample. All conclusions presented in this investigation are based on this principle. Next, we used this method to measure Ang-(1-12) in both human and rodent blood samples. We also verified ex vivo Ang-(1-12) generation in blood samples incubated at 37 °C. Given that Ang II generation from Ang-(1-12) might particularly occur at tissue sites, we also collected tissue samples from mice and rats, focusing on organs (heart, brain, and kidney)^21–23^ with both low and high angiotensin levels, where this pathway has been described to occur. Furthermore, we repeated these measurements in renin knockout mice, which are expected to display elevated angiotensinogen and Ang-(1-12) levels. Finally, we quantified the metabolism of Ang-(1-12) in human plasma samples in the presence of various enzyme inhibitors to establish which enzymes contribute to its metabolism into known angiotensin metabolites.

## METHODS

### Human Blood Samples

Residual blood samples were obtained during a prospective observational study from 6 patients suspected of having COVID-19 (2 women, 4 men; mean age, 56 years; range, 25–76 years).^24^ The samples for the measurement of angiotensins (3 mL) were stabilized in 3 mL of 6 mol/L guanidine hydrochloride (for the measurement of angiotensins), while those for the measurement of renin and angiotensinogen were collected in heparin tubes. The stabilized samples were stored at −70 °C, while heparin plasma was stored at −20 °C.

### Renin Knockout (Ren^−/−^) Mouse Model

We generated a germ-line FVB/N mouse line globally lacking renin expression by crossbreeding C57BL/6 Ren^−/−^ for 8 generations to the FVB/N inbred background. The global renin knockout mouse (Ren^−/−^) with targeted deletion of the *Ren1c* gene, previously generated on the C57BL/6 genetic background,^25^ was a gift from the late Oliver Smithies. By genotyping, we selected mice for breeding that had lost both renin-encoding genes of the FVB/N strain (*Ren1d* and *Ren2*) based on their close proximity on the FVB/N chromosome 1. The wild-type allele of the resulting backcross contains the FVB/N native *Ren1d* and *Ren2* genes. Ren^−/−^ and their wild-type littermates were used for experiments.

### Sprague-Dawley Rats

Rats on the Sprague-Dawley Hannover genetic background strain bred at the animal facility of the Max Delbrück Center for Molecular Medicine, Berlin, were used in this study.

### Animal Experiments

Mice were maintained in individually ventilated cages at a maximum number of 5. Mice cages were kept in a room with a controlled temperature of 22±1 °C under a standard light/dark cycle of 12 hours each. Mice were fed with commercial-standard mouse chow and had access to water ad libitum. All experiments were performed with adult mice at an age between 20 and 22 weeks. In total, 12 mice were used in this study: 6 Ren^−/−^ (3 males and 3 females) and 6 wild-type controls (3 males and 3 females).

Rats were housed individually in ventilated cages in a temperature-controlled room maintained at 22±1 °C with a 12-hour light/dark cycle. They had ad libitum access to tap water and standard commercial rat chow. For sample collection, 3 male Sprague-Dawley wild-type rats aged 16 to 19 weeks were used.

Animal experiments were performed at the animal facility of the Max Delbrück Center for Molecular Medicine, Berlin, following the European Union directive 2010/63/EU for animal experiments. All procedures were previously approved by the Berlin State Office for Health and Social Affairs (Landesamt für Gesundheit und Soziales).

### Animal Sample Collection

Blood was collected from the right ventricle upon death after isoflurane inhalation. Five hundred μL of blood was immediately dispensed and mixed with 5 mL of ice-cold 4 mol/L guanidine thiocyanate solution (Carl Roth No. 0017.1). The remaining blood was dispensed in an EDTA-coated tube for plasma collection after centrifugation at 2000*g* for 10 minutes. Whole brain and left kidney were harvested from mice, and hearts were harvested from rats. Tissues were washed in ice-cold PBS, and then snap-frozen in liquid nitrogen. All samples were stored at −80 °C until LC-MS/MS processing.

### Mouse Plasma Angiotensinogen Quantification by Western Blotting

Plasma angiotensinogen was quantified using an anti-angiotensinogen antibody (1:100; IBL No. 28101) that detects intact and des-Ang I-angiotensinogen. We have previously validated the specificity of this antibody for western blot by using angiotensinogen-KO samples, including plasma.^19,26^ Plasma was diluted in distilled water (1:10, v/v). Total protein levels were quantified with the BCA Assay (Sigma No. BCA1-1KT). Plasma was mixed with the reducing loading buffer based on the Laemmli formulation (1:4, v/v; Carl Roth No. K929.1) and denatured by boiling at 95 °C for 5 minutes. Thirty μg of protein was separated by electrophoresis using a 10% polyacrylamide sodium dodecyl sulfate gel. Proteins were transferred to a nitrocellulose membrane that was blocked (Intercept, LI-COR No. 927-70001) and incubated overnight with the primary anti-angiotensinogen antibody. Angiotensinogen bands were visualized using an Odyssey infrared imaging system and the secondary anti-rabbit conjugated IRDye-800CW antibody (1:10 000; LI-COR No. 926-32213). Angiotensinogen signals were normalized using an anti-transferrin antibody (1:2000; R&D Systems No. AF3987), the bands of which were visualized upon incubation with the secondary anti-goat conjugated IRDye-680RD antibody (1:10 000; LI-COR No. 926-68074) as described above.

### Renin and Angiotensinogen Measurements in Human Plasma

Human renin was measured by a radioimmunometric assay (Cisbio, Saclay, France) and human angiotensinogen by a commercial ELISA (IBL, Hamburg, Germany).

### Metabolism of Ang-(1-12) in Human Plasma

Heparin plasma was obtained from 3 healthy volunteers (1 woman, 2 men; age range 30–40 years). The plasma was incubated for 1 hour at 37 °C, in the absence or presence of the renin inhibitor aliskiren (10 µmol/L), the ACE inhibitor lisinopril (10 µmol/L), the chymase inhibitor chymostatin (10 µmol/L), the aminopeptidase A inhibitor amastatin (10 µmol/L), the neprilysin inhibitor sacubitrilat (LBQ-657, 10 µmol/L), or the prolyl oligopeptidase inhibitor ZPP (20 µmol/L). All inhibitors were from Sigma Aldrich, St. Louis, MO. Next, the plasma was diluted 1:10 in PBS (t=0) and incubated again for 1 hour at 37 °C (t=60) with 1000 ng/mL Ang-(1-12; Sigma Aldrich). Samples at 0 and 60 minutes were collected in 6 mol/L guanidine hydrochloride, and angiotensins were quantified by LC-MS/MS. The purity of the Ang-(1-12) was >99%, but we observed that it contained 0.7% Ang II and 0.1% Ang I, while Ang III (angiotensin-[2-8]), Ang IV (angiotensin-[3-8]), Ang-(1-7), and Ang-(1-5) were undetectable. For this reason, we subtracted the angiotensin levels measured at t=0 from those at t=60 minutes.

### Quantification of Human and Murine Ang-(1-12)

Human (DRVYIHPFHLVI, molecular weight: 1508.8 Da) and murine (DRVYIHPFHLLY, molecular weight: 1572.8 Da) Ang-(1-12) were quantified by LC-MS/MS. To prevent angiotensin degradation in murine tissue during processing, the frozen pieces (30–90 mg) were homogenized using a pestle and mortar filled with liquid nitrogen. Notably, 6 mol/L of aqueous guanidinium chloride supplemented with 1% (v/v) trifluoroacetic acid (Sigma Aldrich, Munich, Germany) was added to the frozen tissue powder to a tissue concentration of 100 mg/mL. The powder was dissolved by cooled sonication using a 2-mm microtip (Sonics and Materials, Newton, NJ). Stable isotope-labeled IS for human and murine Ang-(1-12)–containing isoleucine C^13^/N^15^ (mass +7) at [Ile5] were spiked to tissue homogenates and stabilized blood samples at 1000 pg/mL for internal standardization. The samples then underwent C18-based solid-phase extraction for purification before being subjected to liquid chromatography using a reversed-phase analytical column operating in line with a Xevo TQ-S triple quadruple mass spectrometer (Waters). Two independent MS/MS transitions based on the mass-to-charge ratio of the parent (+3) and fragment ions (+2) were recorded for each analyte and IS simultaneously. Signals from IS were used to correct for peptide recovery in the sample preparation procedure and matrix signal suppression for each analyte in each individual sample. MS/MS transitions were as follows:

Human Ang-(1-12): 503.9>583.7; 503.9>640.2

IS human Ang-(1-12): 506.2>587.2; 506.2>643.7

Murine Ang-(1-12): 525.3>583.7; 525.3>640.2

IS Murine Ang-(1-12): 527.6>587.2; 527.6>643.7

Signals for all 4 analytes were recorded simultaneously in all samples to show the specificity of the gained signals. Figure S1 shows that both human and murine Ang-(1-12) can be detected reliably and independently. The lower limit of quantification was 2 fmol/mL in blood and 10 fmol/g in tissue (ie, 3 pg/mL and 15 pg/g, respectively). Ang I, Ang II, Ang III, Ang IV, Ang-(1-7), and Ang-(1-5) were determined by LC-MS/MS as described before.^27,28^

### Statistical Analysis

Data are provided as individual values and means. Statistical analysis was done by the Student’s *t* test or by ANOVA, followed by post hoc correction according to Dunnett. The half-life t_1/2_ of Ang-(1-12) during its incubation in plasma was estimated based on the formulas ln(C_t_/C_0_)=−kt and t_1/2_=ln2/k, where C_t_ is the concentration after 1 hour, C_0_ is the concentration at t=0, and t=60 minutes.

## RESULTS

### Human Blood Samples

**Figure 1.**
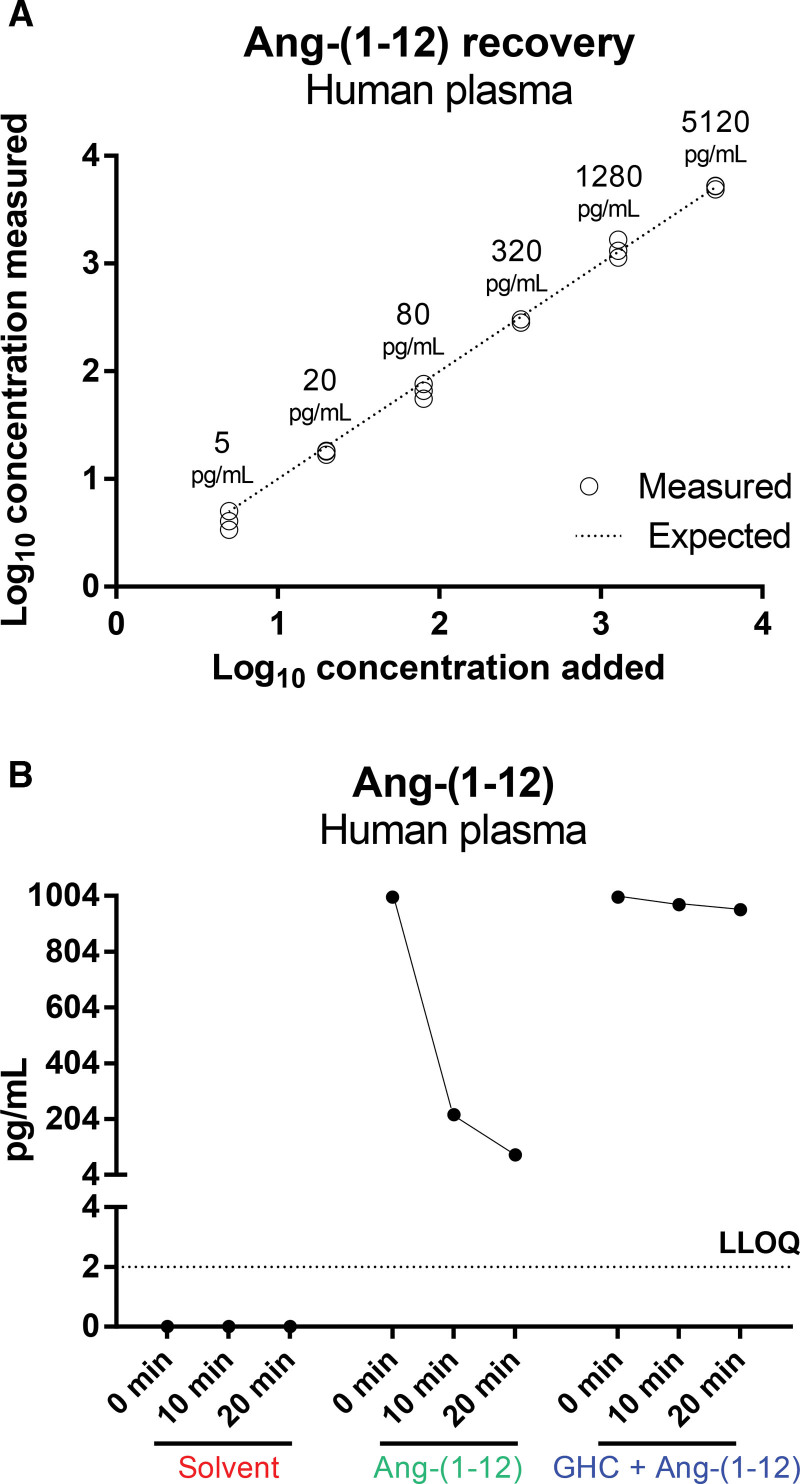
**Recovery of spiked Ang-(1-12).** Spiked angiotensin-(1-12) (Ang-[1-12]) is recovered fully when using the 6 mol/L guanidine hydrochloride collection approach (**A**), but disappears rapidly when using nonstabilized plasma incubated at 37  °C, and cannot be detected in nonstabilized plasma during incubation at 37  °C when no exogenous Ang-(1-12) has been added (**B**). GHC indicates guanidine hydrochloride; and LLOQ, lower limit of quantification.

**Figure 2.**
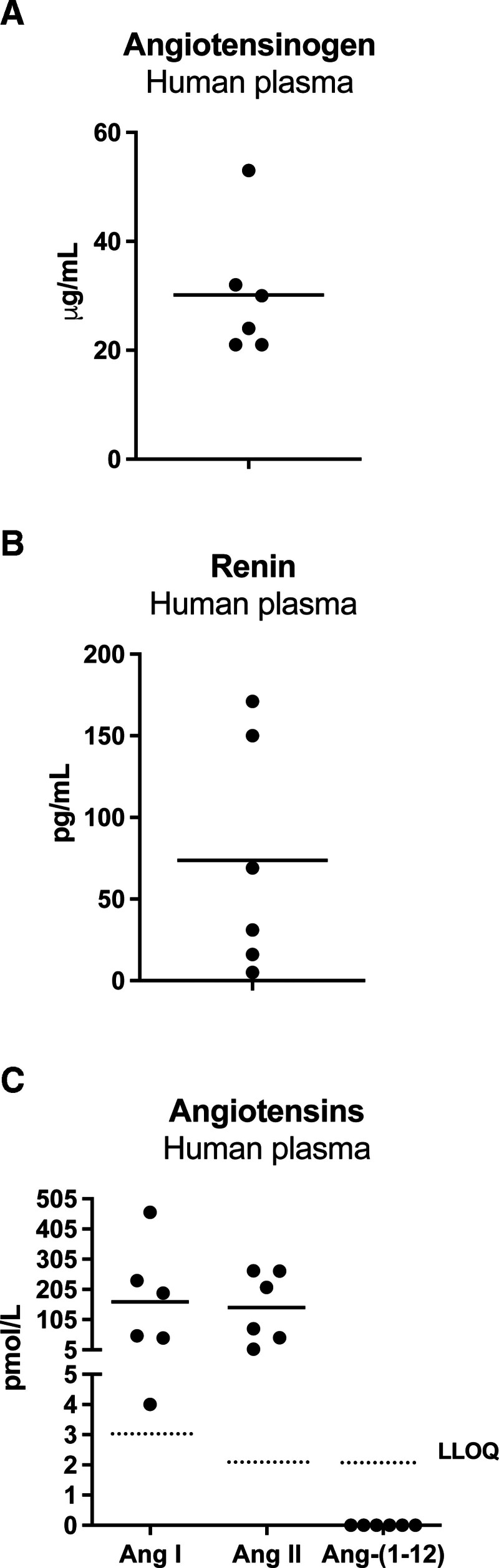
**Renin-angiotensin system components in humans. A** to **C**, Angiotensinogen, renin, angiotensin (Ang) I, Ang II, and Ang-(1-12) levels in 6 humans. Data are individual data points and mean. LLOQ indicates lower limit of quantification.

A rapid collection of blood in 6 mol/L guanidine hydrochloride was chosen for blood collection since this allowed a full recovery of spiked Ang-(1-12; Figure 1A). Importantly, Ang-(1-12) remained undetectable when incubating nonstabilized plasma at 37 °C (Figure 1B), while Ang-(1-12) added to nonstabilized human plasma disappeared almost completely (>80%) within 10 minutes during incubation at 37 °C (Figure 1B). Stabilized human blood samples contained Ang I and Ang II, but Ang-(1-12) was not detectable (Figure 2C). Plasma obtained from the same patients contained normal renin and angiotensinogen levels (Figure 2A and 2B).^29,30^

### Mouse Studies

**Figure 3.**
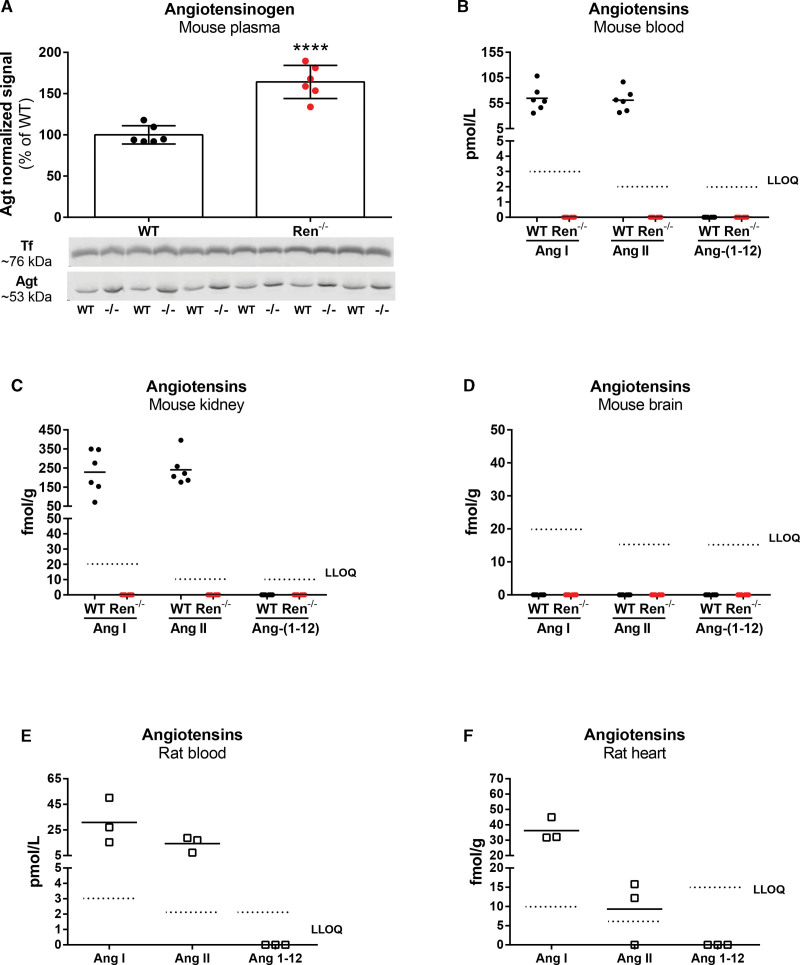
**Renin-angiotensin system components in rats and mice. A**, Angiotensinogen (Agt) in plasma of 6 wild-type (WT) and 6 Ren^−/−^ mice measured by Western blot. ****P<0.0001, Student’s *t* test. **B** to **D**, Angiotensin (Ang) I, Ang II, and Ang-(1-12) in the blood, kidneys, and brains of 6 wild-type and 6 Ren^−/−^ mice. **E** and **F**, Ang I, Ang II, and Ang-(1-12) in the blood and hearts of 3 Sprague-Dawley rats. LLOQ indicates lower limit of quantification; and Tf, transferrin.

Plasma angiotensinogen increased by >50% in Ren^−/−^ mice (Figure 3A). The blood and kidneys, but not the brains, of wild-type mice contained detectable levels of Ang I and II, while Ang-(1-12) was undetectable (Figure 3B through 3D). In Ren^−/−^ mice, all angiotensins, including Ang-(1-12), were undetectable at all sites (Figure 3B through 3D).

### Rat Studies

Ang I and II, but not Ang-(1-12), were detectable in the blood and hearts of Sprague-Dawley rats (Figure 3E and 3F).

### Metabolism of Ang-(1-12) in Human Plasma

**Figure 4.**
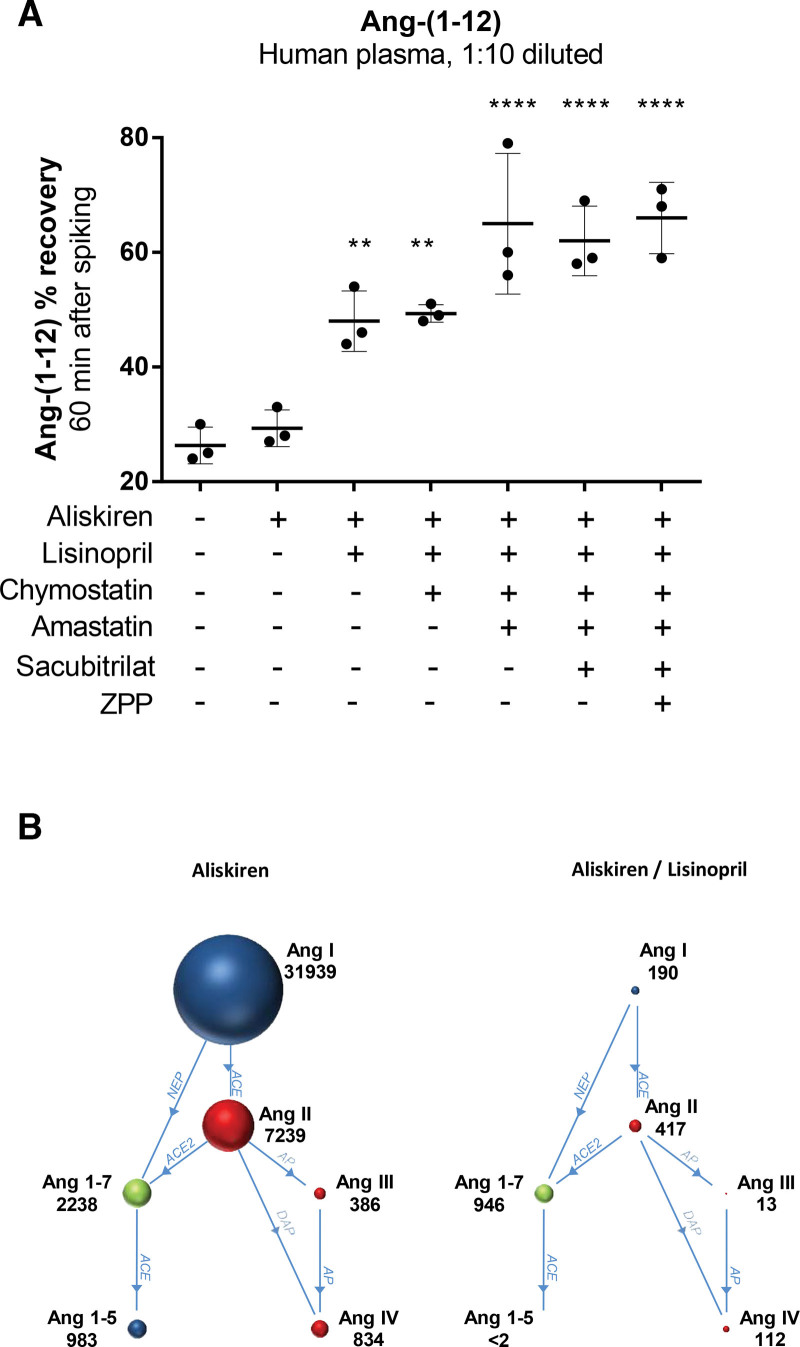
**Metabolism of Ang-(1-12) added to human plasma. A**, Remaining percentage (mean±SD) of angiotensin (Ang)-(1-12) (1000 ng/mL) added to 1:10 diluted human plasma incubated at 37 °C for 60 minutes in the presence or absence of various inhibitors. **P<0.01, ****P<0.001 vs no inhibitors. **B**, Angiotensin metabolites (pg/mL) generated in 1:10 diluted human plasma incubated at 37 °C, following the addition of 1000 ng/mL Ang-(1-12) in the presence of aliskiren or aliskiren + lisinopril. ACE indicates angiotensin-converting enzyme; AP, aminopeptidase; DAP, dipeptidyl aminopeptidase; LLOQ, lower limit of quantification; NEP, neprilysin; and ZPP, Z-Pro-Prolinal.

Adding Ang-(1-12) to 1:10 diluted human plasma samples resulted in its rapid disappearance (estimated half-life of 30 minutes; Figure 4A). As expected, this was unaltered in the presence of aliskiren. The subsequent addition of lisinopril (doubling the half-life) and amastatin (tripling the half-life), but not chymostatin, sacubitril or ZPP, significantly delayed the metabolism of Ang-(1-12). Lisinopril greatly reduced the formation of all known metabolites (Figure 4B), confirming that ACE is responsible for the conversion of Ang-(1-12) into Ang I and II,^31^ and that the formation of Ang III, Ang IV, Ang-(1-7), and Ang-(1-5) also relies on this pathway. However, the reduction in Ang-(1-7) after lisinopril was not as large as that in the other metabolites. Amastatin (nor any of the other inhibitors; data not shown) did not further alter the levels of the 6 angiotensin metabolites quantified here, implying that its effect on the half-life of Ang-(1-12) is due to the formation of an as yet unknown aminopeptidase A metabolite of Ang-(1-12).

## DISCUSSION

This study, making use of a highly sensitive quantification method, does not provide evidence for the presence of intact Ang-(1-12) in humans, rats, or mice, either in blood or tissue. We also did not observe Ang-(1-12) formation when incubating human plasma at 37 °C without inhibitors. This excludes the concept that Ang-(1-12) is formed ex vivo. Furthermore, although angiotensinogen almost doubled in Ren^−/−^ mice, even in such mice, we could not detect Ang-(1-12) nor any other angiotensin metabolite in the blood, kidney, or brain. Finally, we observed that Ang-(1-12), like most angiotensins, is a highly unstable metabolite, with a half-life in the order of <30 minutes in 1:10 diluted human plasma (corresponding to <3 minutes in undiluted plasma and most likely <1 minute in vivo). Ang-(1-12) metabolites in plasma included both Ang II and Ang-(1-7), and thus their effects will be observed when applying Ang-(1-12) either in vivo or to isolated organs. Yet, commercial Ang-(1-12) preparations also contained considerable amounts of Ang II, and this should be taken into account when applying Ang-(1-12).

The measurements in our study differ from earlier work on Ang-(1-12) detection, which relied on the use of Ang-(1-12)–recognizing antibodies (versus murine and human Ang-[1-12]) and included a self-developed radioimmunoassay with a detection limit of 0.75 ng/mL.^11^ A caveat of such assays is that they reflect immunoreactivity, that is, they detect anything that is recognized by the antibody. This might also represent nonspecific binding, including binding to angiotensinogen itself, particularly because its reported cross-reactivity is 3%.^11^ Given that human plasma angiotensinogen levels are in the order of 20 to 60 µg/L, a detection limit of 0.75 ng/mL corresponds to <0.005% of the endogenous concentrations of angiotensinogen. Even when extracting samples before the Ang-(1-12) radioimmunoassay with C18 columns (which retain small molecules only), one has to make sure that the signal that is being picked up does not represent a small fraction of angiotensinogen that is nonspecifically retained by the column. In fact, 0.1% retention would be enough to explain the observed Ang-(1-12) levels. A crucial test here would be to test the antibody in angiotensinogen knockout animals or after angiotensinogen siRNA treatment. However, even that approach cannot rule out background noise that is unrelated to renin-angiotensin system components.

In contrast, LC-MS/MS methods for angiotensin quantification detect peptide fragmentation signatures (mass transitions) that are highly specific for individual angiotensin metabolites without being hampered by background/nonspecific binding issues. This approach also makes use of stable isotope-labeled IS of each individual angiotensin metabolite (added to each sample before preparation), thus ensuring the highest accuracy and specificity. Here, it should be noted that the stable isotope-labeled peptides used in LC-MS/MS are chemically identical to the endogenous target peptides (ie, distinguishing human versus murine) and do not interfere with the final detection of the corresponding endogenous angiotensin metabolites. Using this approach, we reached a lower limit of quantification that was several orders of magnitude below the detection limit of the aforementioned radioimmunoassay. Yet, despite this much greater sensitivity, we were unable to detect intact Ang-(1-12) in the blood of both humans and mice. It has been argued that Ang-(1-12) might particularly occur in tissues, potentially even intracellularly, where it would meet with chymase to allow its conversion to Ang II.^2^ However, we were unable to detect Ang-(1-12) in the kidney and brain, in full agreement with our earlier observations that chymase is an unlikely contributor to Ang II formation in vivo^32,33^ and that angiotensin generation occurs extracellularly.^34^ Furthermore, we repeated all measurements in Ren^−/−^ mice, which, due to the lack of renin, display elevated angiotensinogen levels. Although it has been reported that angiotensin generation continues under such conditions, resulting in even higher tissue concentrations because intact angiotensinogen can now accumulate at much higher levels at tissue sites (subsequently allowing its conversion to Ang-[1-12]),^3^ we were again unable to detect either intact Ang-(1-12) or any angiotensin metabolite in the tissues of Ren^−/−^ mice. This agrees with earlier findings after a bilateral nephrectomy,^4,6^ which suggested that without renin, there is no angiotensin generation. We stress that spiking blood samples with Ang-(1-12) allowed its detection, confirming that we can reliably detect this metabolite. Its detection was complete when the blood had been collected in 6 mol/L guanidine hydrochloride, while without this precaution, around 80% of the added Ang-(1-12) disappeared in 10 minutes.

We subsequently spiked 1:10 diluted human plasma with human Ang-(1-12) and studied during a 60-minute incubation at 37 °C which enzymes are responsible for its metabolism. Here, a serious caveat is that commercial Ang-(1-12) is not 100% pure. In fact, our Sigma preparation of human Ang-(1-12) contained around 0.1% Ang I and 0.7% Ang II. This implies that adding 1000 ng/mL Ang-(1-12) simultaneously yields Ang I and II levels of around 1000 and 7000 pg/mL, respectively. Thus, when, for instance, claiming chymase-independent Ang II formation from Ang-(1-12) added to the mouse aorta, one might consider that this, at least partially, represents Ang II that is already present in the applied Ang-(1-12).^35^ Because of this, we corrected the angiotensin levels measured at t=60 minutes for those that were already found at 0 minutes. When applying inhibitors of renin, ACE, chymase, aminopeptidase A, neprilysin, and prolyl oligopeptidase, we observed that only ACE inhibition and aminopeptidase A inhibition increased the half-life of Ang-(1-12). Furthermore, only ACE inhibition simultaneously suppressed the generation of the known angiotensin metabolites Ang I, Ang II, Ang III, Ang IV, Ang-(1-7), and Ang-(1-5), while aminopeptidase inhibition did not alter the levels of these angiotensins. The latter suggests that its effect on the half-life of Ang-(1-12) is due to the formation of an as yet unknown metabolite of Ang-(1-12). The conversion of Ang-(1-12) by ACE into Ang I and subsequently Ang II is well-known.^23^ Our data confirm that this occurs fully independently of renin. The parallel reduction in Ang III, Ang IV, Ang-(1-7), and Ang-(1-5) along with Ang I and Ang II suggests that their formation also relies on the ACE pathway that involves Ang I and Ang II formation from Ang-(1-12). However, we note that the 50% reduction in Ang-(1-7) was below the 80% to 90% reduction in Ang III, Ang IV, and Ang-(1-5). This likely reflects the fact that the impure Ang-(1-12) preparation contained a considerable amount of Ang II, which can be converted to Ang-(1-7) by ACE2 independently of the presence of Ang-(1-12), while ACE inhibition would prevent its further metabolism to Ang-(1-5). Although direct formation of Ang-(1-7) from Ang-(1-12) by neprilysin has also been demonstrated in renal cortical membranes,^36^ this possibility seems unlikely in our setup, given the low neprilysin levels that occur in blood plasma and the fact that we did not observe a change in Ang-(1-12) recovery after inhibiting neprilysin with sacubitrilat.

## PERSPECTIVE

In summary, our data do not confirm that Ang-(1-12) is an alternative source of endogenous angiotensins and reaffirm renin as the predominant, if not only enzyme–forming, angiotensin. They also imply that Ang-(1-12) immunization^37^ is an unlikely tool to treat cardiovascular diseases, although it might exert effects by neutralizing angiotensinogen itself. However, we currently have an alternative and highly efficient tool to eliminate angiotensinogen: small interfering RNA targeting liver angiotensinogen. This acts by suppressing angiotensinogen synthesis, with 1 injection exerting effects that last 6 months.^18,28,38,39^

## ARTICLE INFORMATION

### Acknowledgments

Images were made with Biorender.

### Sources of Funding

None.

### Disclosures

None.

### Supplemental Material

Figure S1

## Supplementary Material
