# Supplementary material for: Angiotensin-(1-12): Does It Exist? A Critical Evaluation in Humans, Rats, and Mice
Source: Hypertension. 2024 May 8;81(8):1776–84. doi: 10.1161/HYPERTENSIONAHA.124.22856 (PMC11251504; doi:10.1161/HYPERTENSIONAHA.124.22856)
Supplement: Supplementary file 1 [file hyp-81-1776-s001.docx]

**Angiotensin-(1-12): does it exist? A critical evaluation in humans, rats and mice.**

André F. Rodrigues^1,2^, Oliver Domenig^3^, Marko Poglitsch^3^, Michael Bader^1,2,4,5^

and A.H. Jan Danser^6^

^1^Max Delbrück Center for Molecular Medicine in the Helmholtz Association (MDC), Berlin, Germany

^2^German Center for Cardiovascular Research (DZHK), Partner Site Berlin, Germany

^3^Attoquant Diagnostics, Vienna, Austria

^4^Charité Universitätsmedizin Berlin, Berlin, Germany

^5^Institute for Biology, University of Lübeck, Lübeck, Germany

^6^Division of Pharmacology and Vascular Medicine, Department of Internal Medicine, Erasmus MC, University Medical Center Rotterdam, The Netherlands

Correspondence to:

Prof. dr. A.H.J. Danser, PhD
Department of Internal Medicine, Division of Pharmacology and Vascular Medicine, Room Ee-1418b, Erasmus Medical Centre, P.O. Box 2040, 3000 CA Rotterdam, The Netherlands
telephone: 31-10-7043540 ; e-mail: [a.danser@erasmusmc.nl](mailto:a.danser@erasmusmc.nl)

**SUPPLEMENTAL MATERIAL**


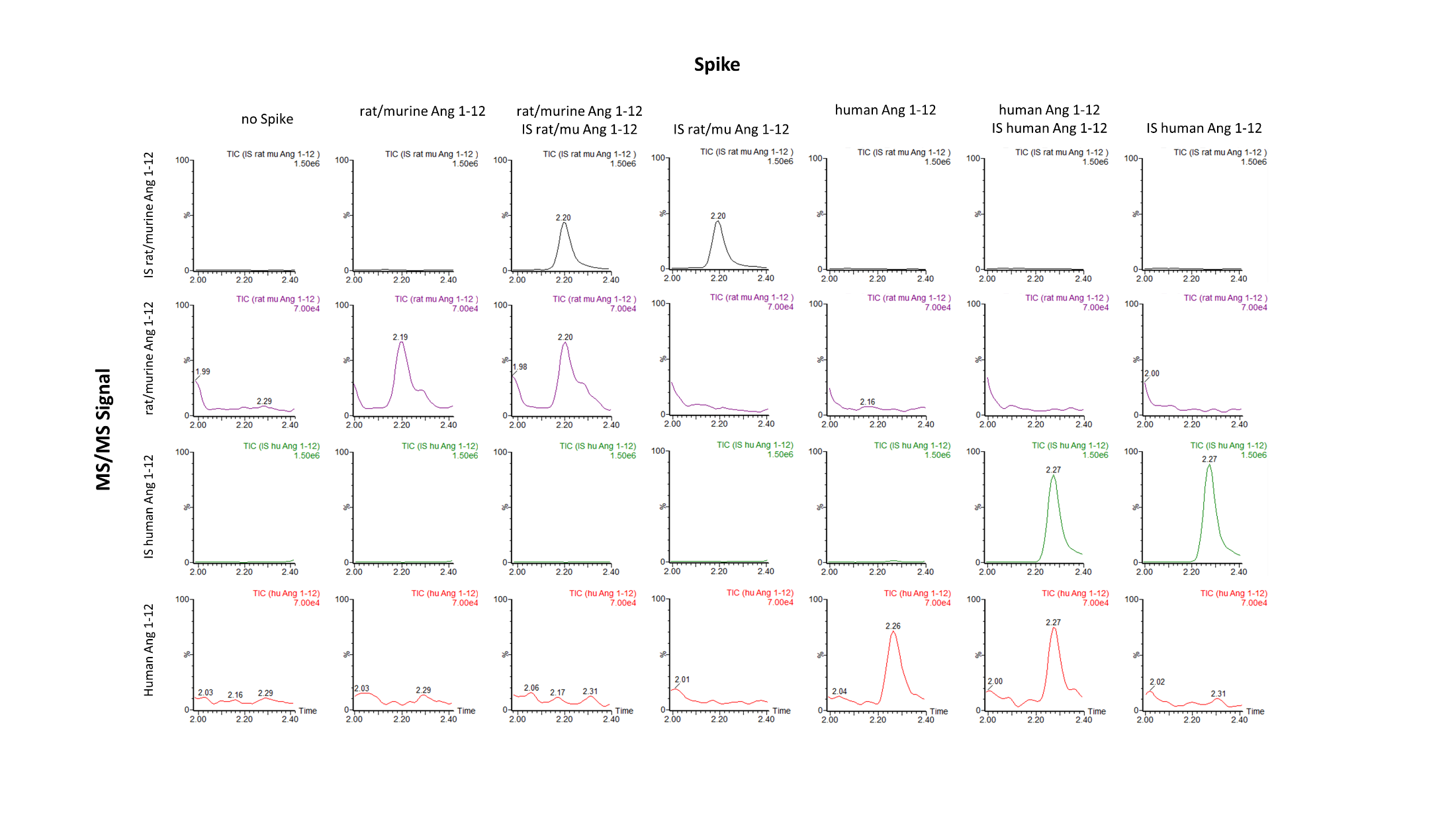


**Figure S1.** Total Ion Counts (percent of the maximum height) of stable isotope-labeled internal standards of human and murine angiotensin-(1-12) (internal standard) as well as the native human and murine angiotensin-(1-12) in regard to the retention time. Value above peak indicates Retention Time. Native angiotensin-(1-12) (100 pg/mL) and internal standards (1000 pg/mL) were spiked to stabilized plasma prior to solid-phase extraction and subsequent LC-MS/MS analysis.
