# Supplementary material for: Angiotensin-(1-12): Does It Exist? A Critical Evaluation in Humans, Rats, and Mice
Source: Hypertension. 2024 May 8;81(8):1776–84. doi: 10.1161/HYPERTENSIONAHA.124.22856 (PMC11251504; doi:10.1161/HYPERTENSIONAHA.124.22856)
Supplement: Supplementary file 2 [file hyp-81-1776-s002.pdf]

*** ARRIVE Reporting Guidelines**

This study involves use of animal models:

Yes

Manuscripts reporting on animal research are expected to adhere to the ARRIVE guidelines. A completed ARRIVE reporting guideline checklist will be required prior to acceptance and the information should be clearly presented in the manuscript. The following shortened version of the checklist should be completed at this time; a completed ARRIVE checklist is required to be uploaded during revision submission.

Animals

Species, age, sex, strain, and sources of animals are described:

Yes

Randomization

Randomization and allocation concealment were performed and described:

Yes

Blinding

Blinding was performed and the process described:

N/A

Inclusions and Exclusions (a)

Specific criteria for inclusions and exclusions are specified:

Yes

Inclusions and Exclusions (b)

Criteria for inclusions and exclusion were set before the study:

Yes

Reporting of Excluded Animals

All animals excluded after the randomization are reported:

N/A

Statistical Methods

Statistical Methods are described:

Yes

Date completed: 04/19/2024 15:16:29

User pid: 1473
